# Determinants of birth asphyxia among live birth newborns in University of Gondar referral hospital, northwest Ethiopia: A case-control study

**DOI:** 10.1371/journal.pone.0203763

**Published:** 2018-09-07

**Authors:** Lisanu Wosenu, Abebaw Gebeyehu Worku, Destaw Fetene Teshome, Abebaw Addis Gelagay

**Affiliations:** 1 Ibex General Private Hospital, Amhara National Regional State, Gondar, Ethiopia; 2 Department of Reproductive Health Institute of Public Health College of Medicine and Health Sciences University of Gondar, Gondar, Ethiopia; 3 Department of Epidemiology and Biostatistics Institute of Public Health College of Medicine and Health Sciences University of Gondar, Gondar, Ethiopia; Aga Khan University, UNITED STATES

## Abstract

**Background:**

Birth asphyxia, which accounts for 31.6% of all neonatal deaths, is one of the leading causes of such mortality in Ethiopia. Early recognition and management of its contributing factors would modify the problem. Thus, this study aimed to identify the determinants of birth asphyxia among live births at the University of Gondar Referral Hospital, northwest Ethiopia.

**Methods:**

A hospital-based unmatched case-control study was conducted from April to July 2017.Cases were newborn babies with an APGAR score of < 7at 5 minutes of birth; controls were newborn babies with an APGAR score of ≥7 at 5 minutes of birth. Every other asphyxiated baby was selected as a case and every 6^th^ non-asphyxiated baby as a control. A pretested structured questionnaire was used to collect data on maternal sociodemographic characteristics. A pretested structured checklist was used to retrieve data on ante-partum, intra-partum, and neonatal factors of both cases and controls. Data were entered using Epi Info 7 and analyzed using SPSS 20. The bivariate logistic regression analysis was used to identify the relation of each independent variable to the outcome variable. Variables with p values of up to 0.2 in the bivariate analysis were considered for the multiple logistic regression analysis. An adjusted odds ratio (AOR) with a 95% CI and p-value of <0.05 was used to identify significant variables associated with birth asphyxia.

**Results:**

In this study, prolonged labor (AOR = 2.75, 95% CI: 1.18, 6.94), cesarean section delivery (AOR = 3.58, 95% CI: 1.13, 11.31), meconium stained amniotic fluid (AOR = 7.69, 95% CI: 2.99, 17.70), fetal distress (AOR = 5.74, 95% CI: 1.53, 21.55), and low birth weight (AOR = 7.72, 95% CI: 1.88, 31.68) were factors which significantly increased the odds of birth asphyxia.

**Conclusion:**

Prolonged labor, cesarean section (CS) delivery, meconium stained amniotic fluid (AF), fetal distress, and low birth weight were the determinants of birth asphyxia. Thus, efforts should be made to improve the quality of intra-partum care services in order to prevent prolonged labor and fetal complications, and to identify and make a strict follow up on mothers with meconium stained amniotic fluid.

## Introduction

The first 28 days of life and neonatal period are the most vulnerable times for a child’s survival[1]. In 2016, 2.6 million children died in the first month of life globally. About one million of them passed away on their first day of life, and more than two thirds (38%) of the deaths were in sub-Saharan Africa[2]. Ethiopia, one of the countries with the highest neonatal mortality in the world, is responsible for 29 deaths per 1,000 live births[3]-over 9 times more than that of highly developed countries, where the rate is3per 1,000 live births[2]. Obstetric causes, notably preterm birth, severe infection, and birth asphyxia continue to account for a large proportion of the deaths[4, 5].

Birth asphyxia, defined as failure to initiate and sustain default breathing at birth[6], is one of the leading and preventable causes of neonatal mortality. Twenty-three percent of the deaths each year around the world[7] and 31.6% in Ethiopia are attributed to birth asphyxia[8]. Studies conducted in Osogbo, Southwestern Nigeria[9], Southern Nepal[10], and Khulna Urban Slum, Bangladesh[11] also suggest that birth asphyxia is responsible for about 23.9%, 30%, and 39% of the deaths, respectively.

The effect of birth asphyxia is not limited only to death but also has a short and long term neurodevelopment sequelae, including cognitive and motor disabilities which are almost untreatable[12, 13]. Different studies showed that the survivors of asphyxia developed hypoxic-ischemic encephalopathy[4, 14, 15], post-traumatic stress disorders[16], neurologic disability[17], low cognitive functions[18], and neurological sequel[19]. A certain experimental study also revealed that nearly 25% of the newborns who survived birth asphyxia developed neurological disorders, such as cerebral palsy and certain neurodevelopment and learning disabilities[20].

Many studies have shown that birth asphyxia is influenced by multiple factors stemming from the ante-partum period. However, factors relating to intrapartum are the predominant risk factors, accounting for 91% of asphyxia[21]. Determinants commonly noted may vary across regions depending on contexts. In countries like Ethiopia, where birth asphyxia is the leading cause of neonatal mortality, recognizing and managing the determinants of the problem early is of a supreme importance to prevent its occurrence, reduce neonatal mortality rate, and improve neonate quality of life. So, identifying the specific determinants in a specific region is mandatory to take appropriate measures in the locality. However, data on the determinants of birth asphyxia are limited in Ethiopia in general and in the study area in particular. Therefore, this study aimed at identifying the determinants of birth asphyxia among newborns delivered at the University of Gondar Referral Hospital, northwest Ethiopia.

## Methods

### Study design and setting

A hospital-based unmatched prospective case-control study was conducted on newborns delivered from April to July 2017, at the University of Gondar Referral Hospital, Maternity Ward. The hospital is located in Gondar city, 727 km to the northwest of Addis Ababa, the capital of Ethiopia. It is one of the largest tertiary public health institutions in Ethiopia, providing preventive, curative, and rehabilitative services to about 5 million people in the catchment. It also provides delivery services 24 hours a day, 7 days a week by mid-wives, interns, residents, and obstetricians who assist about 4000 deliveries annually.

### Study participants

All live newborns after 28 weeks of gestation during the study period were screened for eligibility for the study. Though such cases were not found, the authors proposed to exclude newborns with one or multiple malformations and incompatibility with life, such as hydrops and cyanotic congenital heart defects. In this study, subjects were categorized into cases and controls. Newborn babies with APGAR scores of <7 at 5 minutes were defined as having birth asphyxia, while newborns with APGAR scores of ≥7 at 5 minutes were considered as not having birth asphyxia.

### Sample size determination and sampling techniques

The sample size was calculated by Epi Info 7 software for the unmatched case-control study. A previous study noted that pre-eclampsia was a key predictor of birth asphyxia. Having taken 95% confidence interval, 80% power, 15.9% proportion of controls with pre-eclampsia, an odds ratio of 2.37[22], 2:1 controls to cases ratio, and a 5% non-response rate, the final sample size was273 (91 cases and 182 controls).

By taking a monthly delivery report from the hospital and considering a 13.8% proportion of asphyxiated newborns from a previous study conducted in the hospital in 2013[23], an average of 1400 deliveries and 193 asphyxiated newborns were expected during the data collection. Based on the APGAR score status newborns were categorized as cases and controls. Every other asphyxiated baby was selected as a case, while every 6^th^non-asphyxiated newborn was enrolled as a control.

### Data collection tools and procedures

Both primary and secondary data (chart review) were used. A pre-tested structured interviewer based questionnaire was used to collect data on maternal sociodemographic factors, such as age, marital status, ethnicity, religion, residence, educational, and occupational status. Data on ante partum (parity, ante partum hemorrhage, co-existing obstetric/medical diseases and antenatal visits), intrapartum (duration of labor, fetal presentation, mode of delivery, labor attendant, meconium stained amniotic fluid and premature rupture of membranes), and neonatal related factors (asphyxia, gestational age, birthweight, sex, and birth type) were abstracted using a pre-tested structured checklist from the medical records of pregnant women who gave birth during the data collection period.

The questionnaire was prepared in English and translated to Amharic (local language and retranslated to English to check for inconsistency, but the English version of the checklist was used to retrieve data from the mothers’ medical records. The data collection tool was pre-tested on twenty live births in the same hospital one week prior to the actual data collection period and modifications were made accordingly. Four professional midwives trained for 2 days collected the data. The principal investigator and the supervisor monitored the data collection process on daily basis to ensure data quality and to check for missing information or potential errors.

Birth asphyxia was determined using the components of the APGAR score table. The score comprised such five components as appearance (color), heart rate, grimaces (reflexes), activity (muscle tone), and respiration each of which is given a score of 0, 1, or 2. A score of ≥7 indicated a newborn was not asphyxiated and in a good condition, whereas a low score (<7) indicated a new born with asphyxia[24].

### Operational definitions

**Prolonged labor** was considered when the labor, after the latent phase of first stage of labor, exceeds 12 hours in primigravida or 8 hours in multipara mothers. **Obstructed labor** was considered when the presenting part of the fetus could not progress into the birth canal, despite strong uterine contractions. **Premature rupture of membranes** (PROM) was defined as a rupture of the membrane of the amniotic sac and chorion occurred more than one hour before the onset of labor. **Mal-presentation** was defined as any fetal presentation other than vertex. **Anemia** in pregnant women was defined when the hematocrit level (HCT) was< 33%. Birth weight was classified and defined as normal (2,500 gm or more) or low (<2500 gm).

### Statistical analysis

Data were entered using EPI Info version 7 and analyzed using SPSS version 20. Descriptive statistics, such as means with a standard deviations, frequencies, and percentages were used to describe maternal sociodemographic, ante-partum, intra-partum, and neonatal related factors.

Bivariate logistic regression was conducted to see the correlation of each independent variable to the outcome variable. Variables with p-values of up to 0.20 in the bivariate logistic regression analysis were identified and fitted to the multiple logistic regression analysis to identify the independent effects of each variable to the outcome variable. An Adjusted Odds Ratio with a 95% confidence intervals (CI) was computed to identify the presence and strength of associations, and statistical significance was declared if p < 0.05.

## Results

### Socio demographic characteristics of participants

A total of 270 (90 cases and 180 controls) participants were involved in this study with a response rate of 98.9%. The mean ages of the mothers of cases and controls were 27.8 (SD = 5.2) and 26.9 (SD = 4.9) years, respectively. Moreover, 39 (43.3%) mothers of the cases and 32 (17.8%) of the controls were rural dwellers. Thirty-five (42.0%) mothers of the cases and 38 (19.3%) of the controls had no formal education, while one-third (33.0%) of the mothers of the cases and half of (54.0%) the controls had secondary and above level of education. Of the participants, 58 (64.5%) mothers of the cases and 77 (42.8%) of the controls were housewives (Table 1).

**Table 1:** Socio-demographic characteristics of the mothers of the cases and controls.

Variables	Cases (n = 90)	Controls (n = 180)
	Number (%)	Number (%)
Age in years		
15–17	2(2.3)	8(2.3)
18–34	74(80.7)	154(89.2)
≥35	14(17.0)	18(8.5)
Marital status		
Single	4(4.4)	9(5.0)
Married	86(95.6)	171(95.0)
Ethnicity		
Amhara	90(100.0)	176(97.8)
Tigre	0(0.0)	4(2.2)
Religion		
Orthodox	85(94.4)	164(91.1)
Muslim	5(5.6)	16(8.9)
Residence		
Rural	39(43.3)	32(17.8)
Urban	51(56.7)	148(82.2)
Educational status		
No formal education	35(42.0)	38(19.3)
Primary	24(25.0)	49(26.7)
Secondary+	31(33.0)	93(54.0)
Occupational status		
Government employed	10(11.1)	36(20.0)
Private employed	12(13.3)	36(20.0)
Merchant	10(11.1)	31(17.2)
House wives	58(64.4)	77(42.8)

### Ante-partum related characteristics

Among the total participants, 44(48.9%) mothers of the cases and 99(55.0%) of the controls had <4 ANC visits; 49(56.7%) of the mothers of the cases and 113(62.8%) of the controls were multipara; 12(13.3%) mothers of the cases and only 6(3.3%) of the controls had anemia during pregnancy; 9(10.0%) mothers of the cases and 6(3.3%) of the controls had ante partum hemorrhage during pregnancy.

### Intra-partum related characteristics

Of the total participants, 34(37.8%) of the cases and 29(16.1%) of the controls delivered by induction. Over half, (60%) of the cases and nearly one-third (31.3%) of the controls were born from mothers with history of prolonged labor; 34(37.8%) of the cases and only 15(8.3%) of the controls delivered with CS, and 40(44.4%) of the cases and only one-tenth (10%) of the controls delivered with stained amniotic fluid (Table 2).

**Table 2:** Intra partum related characteristics of the cases and controls.

Variables	Cases (n = 90)	Controls (n = 180)
	Number (%)	Number (%)
Labor attendant		
Intern	14(15.6)	64(35.6)
Midwife	14(15.6)	63(35.0)
Resident	62(68.9)	53(29.4)
Type of labor		
Spontaneous	56(62.2)	151(83.9)
Augmented/induced	34(37.8)	29(16.1)
Obstructed labor		
Yes	18(20.0)	8(4.4)
No	72(80.0)	172(95.6)
Duration of labor		
Normal	36(40.0)	124(68.7)
Prolonged	54(60.0)	56(31.3)
Mode of delivery		
SVD	40(44.4)	154(85.6)
Instrumental	16(17.8)	11(6.1)
CS	34(37.8)	15(8.3)
Amniotic Fluid		
Stained	40(44.4)	18(10.0)
Non stained	50(55.6)	162(90.0)
Mal presentation		
Yes	17(18.9)	5(2.8)
No	73(81.1)	175(97.2)

### Neonatal related characteristics

Out of the total newborn babies, 147 (54.4%) were male and two-thirds (66.7%) of the cases and nearly half (48.3%) of the controls were male. One-fifth (17.8) of the cases and only 11 (6.1%) of the controls were preterm; 22 (24.4%) of the cases and only 9(5%) of the controls had low birth weight. There were 8 (8.9%) twin newborns among cases and 7 (3.9%) among controls (Table 3).

**Table 3:** Neonatal related characteristics of cases and controls.

Variables	Cases (n = 90)	Controls (n = 180)
	Number (%)	Number (%)
Sex		
Male	60(66.7)	87(48.3)
Female	30(33.3)	93(51.7)
Gestational age		
Preterm	16(17.8)	11(6.2)
Term	65(81.8)	152(84.4)
Post-term	9(10.0)	17(9.4)
Birth weight, in milligram		
<2500	22(24.4)	9(5.0)
≥2500	68(75.6)	171(95.0)
Birth type		
Singleton	82(91.1)	173(96.1)
Twin	8(9.9)	7(3.9)
Fetal heart rate, beat per minute		
<100	32(35.6)	9(5.0)
≥100	58(64.4)	171(95.0)

### Determinants of birth asphyxia

The bivariate logistic regression analysis showed that residence, maternal education and occupational status, places of ANC visits, number of pregnancies, ante-partum hemorrhage, type of labor, duration of labor, fetal presentation, mode of delivery, meconium stained amniotic fluid, obstructed labor, gestational age, sex of the new born, birth weight, and fetal heart rate were associated with birth asphyxia. After adjustments for possible effects of confounding variables, prolonged labor, CS delivery, meconium stained amniotic fluid, and low birth weight were significantly and positively associated with birth asphyxia.

The likelihood of developing birth asphyxia among neonates born from mothers with prolonged labor was 2.75 times (AOR = 2.75, 95% CI: 1.18, 6.94) more compared with their counter-parts. Neonates delivered by CS were 3.6 times (AOR = 3.58, 95% CI: 1.13, 11.31) as likely to have birth asphyxia as those born by spontaneous vaginal delivery. Neonates born with meconium stained amniotic fluid were 7.69 times (AOR = 7.69, 95% CI: 2.99, 19.70) as likely to have birth asphyxia as those born without being meconium stained. Neonates born with low birth weight were 7.7 times (AOR: 7.72, 95% CI: 1.88, 31.68) as likely to have birth asphyxia compared to those born with normal weight. Neonates with intra-partum fetal distress had 5.7 times higher risk of experiencing birth asphyxia than those born with normal fetal heart rate (AOR = 5.74, 95% CI: 1.53, 21.55)(Table 4).

**Table 4:** Bivariate and multivariate logistic regression analysis of factors associated with birth asphyxia, University of Gondar referral hospital, Ethiopia, April-July 2017.

Variables	Birth Asphyxia		Crude OR (95% CI)	Adjusted OR (95% CI)
	Asphyxiated (n = 90)	Not asphyxiated (180)		
Residence				
Urban	51(56.7)	148(82.2)	1	1
Rural	39(43.3)	32(17.8)	3.54(2.01, 6.23)	1.16(0.37, 3.67)
Maternal education				
No formal education	35(38.9)	38(21.1)	2.76(1.49, 4.51)	1.81(0.41, 8.04)
Primary	24(26.7)	49(27.2)	1.47(0.78, 2.77)	2.19(0.76, 6.33)
Secondary+	31(34.4)	93(51.7)	1	1
Maternal occupation				
Government employed	10(11.1)	36(20.0)	0.37(0.17, 0.80)	1.15(0.29, 4.51)
Private employed	12(13.3)	36(20.0)	0.44(0.21, 0.93)	0.86(0.25, 3.00)
Merchant	10(11.1)	31(17.2)	0.43(0.19, 0.94)	0.74(0.21, 2.59)
House wives	58(64.4)	77(42.8)	1	1
Parity				
1(Primipara)	41(45.6)	67(37.2)	1.79(1.01, 3.18)	1.45(0.57, 3.76)
2-4(Multipara)	20(22.2)	28(15.6)	2.09(1.03, 4.27)	1.59(0.41, 6.16)
≥5(Grand multipara)	29(32.2)	85(47.2)	1	1
APH				
Yes	9(10.0)	6(3.3)	3.22(1.11, 9.36)	2.39(0.48, 11.94)
No	81(90.0)	174(96.7)	1	1
Pregnancy Induced Hypertension				
Yes	10(62.5)	6(3.3)	3.63(1.27, 10.32)	3.24(0.69, 15.27)
No	80(31.5)	174(96.7)	1	1
Types of labour				
Spontaneous	56(62.2)	151(83.9)	1	1
Induced	34(37.8)	29(16.1)	3.16(1.77, 5.66)	2.01(0.76, 5.28)
Fetal Presentation				
Cephalic	73(81.1)	175(97.2)	1	1
Not cephalic	17(18.9)	5(2.8)	8.15(2.90, 22.92)	3.29(0.63, 17.21)
Mode of delivery				
SVD	40(44.4)	154(85.6)	1	1
Instrumental	16(17.8)	11(6.1)	5.60(2.41, 13.01)	1.68(0.44, 6.42)
Cesarean section	34(37.8)	15(8.3)	8.73(4.33, 17.57)	3.58(1.13, 11.31)*
Duration of labor				
Normal	36(40.0)	124(68.9)	1	1
Prolonged	54(60.0)	56(31.1)	3.24(1.91, 5.48)	2.75(1.18, 6.94)*
Meconium stained AF				
Yes	40(44.4)	18(10.0)	7.20(3.80, 13.66)	7.69(2.99, 19.70)*
No	50(55.6)	162(90.0)	1	1
PROM				
Yes	35(38.9)	25(13.9)	3.95(2.17, 7.18)	2.24(0.89, 5.58)
No	55(61.1)	155(86.1)	1	1
Obstructed labor				
Yes	18(20.0)	8(4.4)	5.38(2.24, 12.92)	1.63(0.41, 6.42)
No	72(80.0)	172(95.6)	1	1
Birth Attendant				
Intern doctor	14(15.6)	4(35.6)	0.19(0.09, 0.37)	0.72(0.23, 2.16)
Midwife	14(15.6)	63(35.0)	0.19(0.10, 0.38)	0.64(0.20, 2.01)
Resident doctor	62(68.8)	53(29.4)	1	1
Sex of new born				
Male	60(66.7)	87(48.3)	2.14(1.26, 3.62)	1.92(0.84, 4.390
Female	30(33.3)	93(51.7)	1	1
Gestational age				
Pre-term	16(17.8)	11(6.1)	3.40(1.50, 7.73)	1.17(0.29, 4.67)
Term	65(72.2)	152(84.5)	1	1
Post-term	9(10.0)	17(9.4)	1.24(0.53, 2.92)	1.82(0.54, 6.06)
Birth weight				
Normal	68(75.6)	171(95.0)	1	1
Low	22(24.4)	9(5.0)	6.15(2.69, 14.03)	7.72(1.88, 31.68)*
Fetal heart rate, in bpm				
<100	32(35.6)	9(5.0)	11.0(4.96, 24.38)	5.74(1.53, 21.55)*
≥100	58(64.4)	171(95.0)	1	1
Birth type				
Twin	8(8.9)	7(3.9)	2.41(0.85, 6.88)	0.78(0.13, 4.65)
Singleton	82(91.1)	173(96.1)	1	1

* Statistically significant at P<0.05, COR (Crude Odds Ratio), AOR (Adjusted Odds Ratio)

## Discussion

Asphyxia is the leading cause of mortality due to hypoxic-ischemic damage to newborns. Hence, the quality of medical care at birth is crucial to reduce the overall newborn mortality and its long-term consequences. In this study, it was attempted to identify the determinants of birth asphyxia among newborns at the University of Gondar Referral Hospital. Prolonged labor, CS delivery, meconium stained amniotic fluid, fetal distress, and low birth weight were identified as determinants of birth asphyxia.

Prolonged labor is most likely to occur if a woman’s pelvis is not large enough for her baby’s head to pass through, or if her uterus does not contract sufficiently, or if there is a slow effacement of the cervix[25, 26]. In this study, newborns who were born from mothers with prolonged labour had 2.75 times more risk for birth asphyxia. A previous study at the University of Gondar Referral Hospital, Ethiopia, noted similar findings[23]. Our finding was also consistent with those studies conducted at Dhaka Medical College Hospital[19], Cameroon[27], Gusau, Nigeria[28], Hospital Universitario del Valle, Colombia[29], Port Moresby General Hospital[30], Stockholm and Gotland, Sweden[31] where the odds of birth asphyxia among babies born to mothers with prolonged labor were about two to sixteen times higher than those born to mothers who had no prolonged labor. This could be due to the fact that if the labor does not progress normally, the woman may experience serious complications, such as dehydration, exhaustion, or rupture of the uterus[32]. It is also clear that when labor is prolonged, there is a high probability for the fetus to become distressed. All these complications can lead to birth asphyxia. Prolonged labor may also contribute to maternal infection[33, 34], hemorrhage[35, 36], and neonatal infection[34] which might cause the newborns to develop birth asphyxia.

Newborns delivered by cesarean section had 3.6times more risk for birth asphyxia. This finding was consistent with those of studies conducted in Gusau, Nigeria[28], Combined Military Hospital Multan, Pakistan[13], Dr Soetomo Hospital Surabaya, Indonesia[22], and Vali-eAsr hospital, Tehran, Iran[37] where the odds of birth asphyxia among newborns delivered by CS were about 2 to 19 times higher compared to newborns who were born through spontaneous vaginal delivery. The high rate of asphyxia among newborns delivered by CS might be due to the fact that either most of the mothers came with complications or the decision for CS might be made late after they develop complications or due to factors associated with indications of caesarean section and an added stress of anesthesia[38]. The fetus chest might be squeezed when the newborn passes through the birth canal in vaginal delivery which might evacuate secretion. This in turn reduces the chance of developing birth asphyxia, but this physiological advantage is not seen in CS delivery.

Neonates born from mothers with history of meconium stained amniotic fluid had 7.69 times more risk for birth asphyxia. This finding was consistent with those of previous studies conducted at the University of Gondar Referral Hospital[23], Mulago Hospital, Uganda[39], India[40], and on Swedish Urban Population[41]. The possible reason could be that meconium stained AF results in peripartum inhalation of meconium-stained amniotic fluid, leading to chemical pneumonitis with inflammation of the pulmonary tissues, mechanical obstruction of airways, and pulmonary air leak, inducing hypoxia[42].

Another neonatal factor found to have significantly associated with birth asphyxia was intra-partum fetal distress. Neonates who had intra-partum fetal distress had about 5.74 times higher risk of developing birth asphyxia. A similar result was obtained in a previous study done in this hospital[23] and other studies conducted at Al-Diwaniya Maternity and Children Teaching Hospital[43]. The possible explanation is that fetal distress is the main indication of emergency CS which is a known risk factor for birth asphyxia.

Low birth weight was also an important determinant of birth asphyxia in this study. Neonates who had low birth weight were 7.72 times more likely to have birth asphyxia than those who had normal birth weight. This finding was in line with those of studies conducted at the University of Gondar Referral Hospital[23, 44], Mulago Hospital, Uganda[39], Dr Soetomo Hospital Surabaya, Indonesia[22], Vali-eAsr Hospital, Tehran-Iran[37], Civil Hospital in Karachi, Pakistan[45], and Pattani Hospital, Thailand[46]. This could be explained by the fact that a high proportion of small babies might be pre-term that they might not have enough surfactant which might lead to suffering from difficulty of breathing and developing difficulty in cardiopulmonary transition and subsequent birth asphyxia.

## Conclusions

In this study, the determinants of birth asphyxia are mainly related to the duration of labor, fetal conditions and bad fetal outcomes, and the mode of delivery. Prolonged labor, meconium stained AF, CS delivery, fetal distress, and low birth weight were the determinants of birth asphyxia. Therefore, efforts should be made to improve the quality of intra-partum care service in order to prevent prolonged labor and fetal complications, and to identify and make a strict follow up of mothers with meconium stained amniotic fluid.

## Ethical approval and consent to participate

Ethical clearance was obtained from the Ethical Review committee of the Institute of Public Health, College of Medicine and Health Sciences, the University of Gondar. A permission letter was obtained from the Chief Clinical Officer of the University of Gondar Referral Hospital. Informed verbal consent was also obtained from each study participant to review and use their medical records (charts). Participation in this study was voluntary. Confidentiality of information obtained was ensured using code numbers rather than names and keeping the questionnaire and checklist safe throughout.

## Supporting information

S1 Data This is the data set of the study. (SAV)

## Abbreviations

AF: Amniotic Fluid
ANC: Ante Natal Care
AOR: Adjusted Odds Ratio
APGAR: Appearance Pulse Grimace Activity Respiration
APH: Ante Partum Hemorrhage
CI: Confidence Interval
COR: Crude Odds Ratio
CS: Cesarean Section
PROM: Premature Rupture of Membrane
SPSS: Statistical Package for Social Sciences
